# The psychological effects of forced family separation on asylum-seeking children and parents at the US-Mexico border: A qualitative analysis of medico-legal documents

**DOI:** 10.1371/journal.pone.0259576

**Published:** 2021-11-24

**Authors:** Kathryn Hampton, Elsa Raker, Hajar Habbach, Linda Camaj Deda, Michele Heisler, Ranit Mishori

**Affiliations:** 1 Physicians for Human Rights, New York, NY, United States of America; 2 Formerly Physicians for Human Rights, New York, NY, United States of America; 3 Department of Internal Medicine, University of Michigan Medical School, Ann Arbor, MI, United States of America; 4 Department of Health Behavior and Health Education, School of Public Health, University of Michigan, Ann Arbor, MI, United States of America; 5 Department of Family Medicine, Georgetown University Medical Center, Washington, DC, United States of America; Brown University, UNITED STATES

## Abstract

The U.S. government forcibly separated more than 5,000 children from their parents between 2017 and 2018 through its “Zero Tolerance” policy. It is unknown how many of the children have since been reunited with their parents. As of August 1, 2021, however, at least 1,841 children are still separated from their parents. This study systematically examined narratives obtained as part of a medico-legal process by trained clinical experts who interviewed and evaluated parents and children who had been forcibly separated. The data analysis demonstrated that 1) parents and children shared similar pre-migration traumas and the event of forced family separation in the U.S.; 2) they reported signs and symptoms of trauma following reunification; 3) almost all individuals met criteria for DSM diagnoses, even after reunification; 4) evaluating clinicians consistently concluded that mental health treatment was indicated for both parents and children; and 5) signs of malingering were absent in all cases.

## Introduction

Reports, beginning in 2017, that the Trump administration was separating young migrant children–as young as four months old [1]—from their parents led to a nationwide outcry. The U.S. government separated more than 5,000 children between 2017 and 2018 through its “Zero Tolerance” policy that aimed to deter asylum seekers from entering the United States through the U.S.-Mexico border. Following litigation, the practice was halted by the courts, but the government was unprepared to reunite parents with their children, many of whom had been sent to states throughout the country to stay in shelters or with foster families. Jonathan White, commander of the U.S. Public Health Service Commissioned Corps, who at the time was a deputy director for children’s programs at the Department of Health and Human Services’ (HHS) Office of Refugee Resettlement and a career public health official at the HHS, testified before a House panel in July 2018 that he had warned the administration that forced separation would cause “traumatic psychological injury” [2]. Medical consultants for the Department of Homeland Security, through the Senate whistleblower protection program, also warned the administration of the trauma that would be caused by family separation [3]. Nevertheless, the administration proceeded to instruct government employees to separate families while denying that there was a formal family separation policy in place. A report by the Office of Inspector General of the Department of Justice states that the Attorney General and other senior officials indeed knew that prosecutions would result in separations [4]. In February 2021, the Biden Administration created a new Interagency Task Force on the Reunification of Families in order to coordinate reunification of the separated families, but as of August 1, 2021, the Task Force reported that only 42 children had been reunified, and at least 1,841 children were still separated from their parents [5].

The question of whether the separation caused lasting trauma has been a matter of political debate. Former President Trump, for example, claimed in a presidential debate, “[The children are] fine, the facilities they were in were so clean” [6]. However, the scientific literature, and virtually all experts agree that experiencing such trauma can have persistent effects. Such childhood exposures are also known as Adverse Childhood Events, or ACEs [7]. ACEs are linked with disruption of neurodevelopment and with negative effects on social, emotional, and cognitive functioning [8, 9]. ACEs have also been associated with negative intergenerational effects [10]. High levels of extreme or repetitive stress are correlated with increased risk of mental health conditions, such as depression and PTSD, and even physical conditions such as cancer, stroke, diabetes, and heart disease [8]. Children who experienced trauma often have sleeping difficulties and exhibit heightened responses to perceived threats, in the form of crying, being fearful, or clinging to a trusted adult. Aggressive behaviors are also common, as is regression, defined as reverting to an earlier developmental stage. Many families and adults arriving at the U.S. border have already experienced significant pre-migration trauma in their home countries [11]. This trauma is further compounded by subsequent trauma through their experience in U.S. immigration detention, which has been linked with deterioration in mental health symptoms [12], similar to the experience of refugees in other countries which have punitive immigration detention systems, such as Australia [13].

Individual health professionals and numerous medical organizations actively opposed the policy of family separation due to the trauma that it was predicted to cause [14]. The American Academy of Pediatrics called it “government-sanctioned child abuse” [15], and Physicians for Human Rights determined that the harms documented are consistent with the legal definition of torture and temporary enforced disappearance under international human rights law [16].

Due to the difficulty to access people while they are detained and after they are deported, very little empirical research has been published on the psychological effects of the Zero Tolerance forced separation policy on those directly affected. A study conducted in South Texas Family Residential Center, a detention center where U.S. Immigration and Customs enforcement detains parents and minor children together, completed 425 interviews with mothers and evaluations of 150 children, of which 17 percent had been forcibly separated under the policy. The study revealed that children who had been separated had worse outcomes compared to those who had not been separated, including higher rates of emotional problems (49% vs. 29%, p = 0.003) and greater total difficulties (15% vs. 9%, p = 0.015) [17]. These findings are consistent with scientific literature from the United States and other countries regarding the traumatic impact of family separation on refugee and asylum-seeking children, including higher rates of PTSD and depressive disorders which can continue into their adulthood and contribute to lower academic achievement, attachment difficulties, and poor mental health [18–22].

To better understand the psychological effects of family separation on those directly affected, we systematically examined narratives obtained by trained clinical experts who interviewed and evaluated parents and children after reunification as part of a medico-legal process. These evaluations were conducted for various types of legal cases, including the clients’ asylum claims, appeals for clients who did not pass their credible fear interviews, and lawsuits for damages related to family separation.

## Methodology

From July 26, 2018, through December 14, 2019, clinician members of the Physicians for Human Rights U.S. Asylum Program referred 818 individual evaluations to clinicians for the purpose of providing medico-legal affidavits, out of which 42 evaluations were conducted for parents or children who had been forcibly separated by the U.S. government. The clinicians evaluating the separated family members included psychologists (ten), psychiatrists (six, including one child psychiatrist), pediatricians (two), social workers (two), a family physician (one) and a licensed professional counselor (one). In total, nine were men, 13 were women.

Out of 42 completed affidavits, 11 medico-legal affidavits were excluded from the study because the evaluation focused on the asylum case without addressing the family separation and subsequent harms that occurred in the United States. The researchers analyzed the remaining 31 affidavits by performing a content analysis to identify themes and sub-themes through open, axial, and selective coding [23, 24]. The coding tool was jointly developed by the research team, using Dedoose, a qualitative analysis software program. Data reached code saturation by the second intercoder agreement trial and meaning saturation by the fourth intercoder agreement [25]. After four trials, the research team established a 78 percent intercoder reliability agreement. An experienced qualitative researcher conducted a peer audit of the coding. Through an iterative and consensus-based process, the research team revised themes and sub-themes. The University of Michigan Institutional Review Board (IRB) reviewed the research plan and designated it as exempt from full IRB review since the data are anonymized and thus this human subject study presents no greater than minimal risk to participants.

## Results

### Demographics

The medico-legal affidavits involved 25 family separation cases, comprising a total of 31 individuals, including five parent/child pairs and a husband/wife pair (the husband was separated from their daughter at the U.S. border, the wife migrated afterwards with their son). Most of the parents and children (27/31) were reunited at the time of evaluation. Three children were not reunited with parents at the time of the evaluation; two of the children’s parents were deported and another child was separated from his father who was in U.S. immigration detention at the time of the evaluation. Most of the parents and children had been released from detention into community settings at the time of the evaluation (24/31); six of the mothers were evaluated in an immigration detention center (South Texas Family Residential Center) and one child was in custody of the U.S. Office of Refugee Resettlement. Other demographics in Table 1.

**Table 1:** Demographic statistics related to the parents and children.

Dimension	*n*
**Country of origin** *	
Honduras	13
Guatemala	9
El Salvador	8
**Parent age ranges**	
Female (24–45) [27]	15
Male (32–40)	4
**Children age ranges**	
Female (6–14)	4
Male (6–17)	8
Children < age 10	8

* In one sensitive case of political persecution, the country of origin was redacted.

### Major themes in the clinicians’ assessments

The following five categories emerged from the data analysis: 1) parents and children shared similar pre-migration traumas and the event of forced family separation in the U.S.; 2) parents and children reported signs and symptoms of trauma following reunification; 3) almost all individuals met criteria for DSM diagnoses, even after reunification; 4) evaluating clinicians consistently concluded that mental health treatment was indicated for both parents and children; and 5) that signs of malingering were absent in all cases.

### Migration trigger and separation event as reported by parents and children

Due to targeted acts of violence in their home countries, all parents arrived at the U.S. border having already been exposed to significant trauma. Many were victims of gang-based persecution including death threats, physical assault, murder of relatives, extortion, sexual assault, and/or robbery. All parents expressed fear that their child would be harmed or killed if they stayed within their home country. In almost all cases, their children also had experienced severe harm before fleeing; gang members drugged, kidnapped, poisoned, and threatened children, including threats of death, violence, and/or kidnapping if they or their parents did not comply with the gang’s demands. Parents were confident that the journey to the United States would ensure protection for their children after failed attempts to evade gang-based persecution in their home country.

According to the reports of the parents and children, the conduct of U.S. officials was punitive rather than protective. When parents arrived in the United States, they reported that immigration authorities forcibly removed children from their arms and transferred parents to other facilities while their children slept. In some cases, the children “disappeared” while their parents were in court rooms or receiving medical care. Almost all reported that immigration authorities failed to provide any explanation as to why they were being separated, where their family members were being sent, and if / how they would be reunited. In addition, the asylum narratives documented instances of four parents who were taunted and mocked by immigration authorities when asking for the whereabouts of their children. Half of the parents interviewed by PHR clinicians reported poor conditions at the detention facilities where they were held, and the children also reported being mistreated or living in poor conditions while detained and while in foster care.

### Diagnoses observed and recorded

The mental health conditions diagnosed by the evaluating clinicians and depicted in the medico-legal affidavits were found to be highly consistent with the parents’ and children’s reports of their traumatic experiences in detention and family separation. At the same time, several clinicians commented on the likelihood that the separation exacerbated pre-existing trauma from events and incidents in their home country. According to the clinicians, most individuals met diagnostic criteria for at least one mental health condition such as post-traumatic stress disorder (PTSD), major depressive disorder (MDD), or generalized anxiety disorder (GAD); see Table 2. While several people did not meet all diagnostic criteria for these conditions, nearly everyone exhibited hallmark features and symptoms of these three major conditions. It is worth noting that two children evaluated long after reunification with their parents still exhibited severe symptoms and, despite some symptom improvement, still met criteria for diagnoses at the time of evaluation; a six-year-old girl from Guatemala met criteria for PTSD one year after reunification with parents and an 8-year-old boy met criteria for PTSD and separation anxiety disorder two years after reunification with parents. Neither had exhibited these symptoms prior to the separation event.

**Table 2:** Describes the conditions diagnosed by PHR medical experts.

Gender	Age	Country of Origin	Diagnoses
**Psychiatric Diagnoses in Adults (16 out of 19)**			
Male	33	Honduras	Criteria not met for DSM-V diagnoses (but patient exhibited several symptoms suggestive of trauma)^#^
Female	Adult*	Guatemala	PTSD, MDD with anxious distress
Female	45	El Salvador	PTSD
Female	29	Guatemala	PTSD, MDD
Female	30	Honduras	PTSD
Female	27	Guatemala	Complex PTSD
Female	24	[Redacted]	Features of PTSD, MDD, GAD, no official diagnoses
Female	Adult*	El Salvador	PTSD
Female	29	Honduras	MDD, features of PTSD, GAD
Female	24	Honduras	MDD, PTSD
Female	26	Honduras	PTSD, MDD, recurrent, severe
Male	32	Honduras	PTSD, features of GAD, MDDψ
Female	28	El Salvador	PTSD
Female	39	Honduras	PTSD, MDD, recurrent and severe
Male	40	El Salvador	PTSD
Female	Adult*	Honduras	PTSD
Male	36	El Salvador	Criteria not met for DSM-V diagnoses (but patient exhibited several symptoms suggestive of trauma)
Female	35	Guatemala	PTSD
Female	36	Guatemala	PTSD, MDD
**Psychiatric Diagnoses in Children (12 out of 12)**			
Male	9	Honduras	PTSD
Male	17	El Salvador	PTSD
Male	8	Honduras	PTSD, MDD recurrent, moderate
Female	6	Guatemala	PTSD, separation anxiety disorder, Depression
Female	7	El Salvador	separation anxiety disorder
Male	8	Honduras	PTSD
Male	6	Guatemala	PTSD, split personality features, anxiety, depression
Male	8	Honduras	Depression, anxiety
Female	6	Guatemala	PTSD
Male	16	Guatemala	PTSD
Female	14	El Salvador	PTSD
Male	8	Honduras	PTSD, separation anxiety disorder

* Ages of three mothers were redacted at their attorneys’ request.

# The client continues to have difficulty thinking about the separation from his daughter, avoids speaking about it, becomes visibly emotional and tearful when he does and is fearful they will be separated again, but was reluctant to speak about his own difficulties, wanting to focus on his family’s needs. The evaluator pointed to multiple resilience factors which are attributed to his psychological wellbeing, including his commitment to his family, his religious faith and his realistic optimism about the future.

ψ The client was described as “overtly distressed and tearful on several occasions when describing past experiences” but appeared to “minimize the severity of his symptoms, as evidenced by the mood scores he provided, which were not always consistent with his affect during the course of the evaluation”. The evaluator attributed this discrepancy to “his longstanding limited insight, which is most likely due to his education, social, and cultural backgrounds”.

### Signs and symptoms of trauma

PHR clinicians identified symptoms and behaviors consistent with trauma and its residual effects in nearly all of the parents and children. These symptoms were present at the time of the family separation as well as the time of the examination post-reunification. Chief concerns included feelings of confusion, general upset to severely depressed mood, constant worry/preoccupations, frequent crying, difficulty sleeping, difficulty eating (loss of appetite), recurring nightmares, and overwhelming anxiety. The asylum-seekers also reported physiological manifestations of anxiety and panic (racing heart, shortness of breath, and headaches) as well as experiencing “pure agony,” emotional and mental despair, hopelessness, and being “incredibly despondent”.

Trauma exposure in adults can manifest physically as well as psychologically, emotionally, and spiritually. Common signs of trauma include lethargy, fatigue, poor concentration, a racing heartbeat, bouts of anxiety, panic attacks, depression, or vague somatic symptoms (e.g., headaches, abdominal pain, general pain). Three of the 19 parents also experienced suicidal thoughts while separated from their children.

The evaluating clinicians noted that the children exhibited reactions that included regression in age-appropriate behaviors, such as crying, not eating, having nightmares and other sleeping difficulties, excessive parental attachment, clinging to caregivers, urinary incontinence, and recurring feelings of fear following reunification with their parents.

### Follow up treatment recommendations

In almost every case encountered, expert evaluators noted that the trauma suffered by the parents and the children were causing significant distress and ongoing functional impairment, requiring further intervention and robust therapeutic support. The interventions most frequently recommended included “trauma-focused psychotherapy,” removal from detention, and psychiatric medications.

The clinicians also commented that a return to their country of origin would lead to exacerbated symptoms due to “returning to the site of initial traumas” and a lack of mental health resources and services available. One clinician said of a mother from Honduras, “Given Ms. X’s lack of access to mental healthcare in Honduras, this degeneration could cause significant morbidity and mortality, as well as incalculably impair the development of her daughter, who may have significant psychiatric trauma due to the recent forced separation from her mother”.

In many cases, symptoms and distress continued after reunification, prompting the evaluators to recommend therapy, and also removal from detention for those still in immigration detention centers. Of a Guatemalan mother, “Her symptoms are expected to continue until she is removed from detention, which is a constant reminder of the trauma of the separation from her sons, and receives appropriate, trauma-focused psychotherapy…. She needs to be in an environment that does not constantly remind her of the trauma of the separation. It is my professional recommendation that she and her sons be released from detention and treated with trauma-focused therapy in the US.” Of a 30-year-old mother, the clinician noted: “The presence of immigration officers is a constant reminder of the trauma she experienced at the hands of immigration officers at the border”.

Clinicians also recommended a number of trauma-informed adaptations which could be made during the on-going legal process. They recommended providing additional time to process questions and formulate responses, repeating questions, pre-medicating with anti-anxiety medication, allowing for frequent breaks to rest and allowing their children to remain with them during the interview or allowing them breaks to see their children. A forensic psychologist said of a 36-year-old mother, “Court officials must use simple language, monitor her understanding, and rephrase material as needed.” A psychiatrist commented regarding another mother’s information processing difficulties due to the trauma of separation from her child, “Symptoms of her disability may interfere with her ability to attend to interactions and to process information”.

The examining clinicians recommended that many of the adults and children receive professional mental health support because, as was stated regarding one of the children, “if left untreated… (he) would be at high risk for future psychological and physical problems”.

### Clinician assessment of credibility

PHR’s experts who evaluated the parents and the children noted that all the interviewed individuals had “demonstrated appropriate emotional reactions to stressful and traumatic situations,” and did not show any signs of malingering, which is described in the DSM-V as “the intentional production of false or grossly exaggerated physical or psychological problems” that are motivated by external incentives. Following in-depth evaluations, which often lasted more than three hours, clinicians uniformly deemed the parents and children as credible historians, showing “no evidence of exaggeration or deception,” providing an account that “constitutes an entirely expectable, natural and cohesive psychological story,” having “no indication of exaggerating or faking symptoms,” and “displayed none of the cardinal features of the malingering patient”.

## Discussion

Our analysis of 31 medico-legal affidavits of parents and children directly affected by forced family separation shows nearly uniform negative mental health outcomes. To our knowledge, this is the first qualitative analysis of the mental health effects of the “zero tolerance” policy, as assessed during in-depth interviews by experienced clinicians.

Physicians for Human Rights and other experts have concluded that the U.S. government’s treatment of parents and children through the policy of family separation constitutes cruel, inhumane, and degrading treatment [21, 26, 27]. Moreover, all cases reviewed for this project rise to the level of torture, defined by the United Nations Convention Against Torture as an intentional act which causes severe physical or mental suffering for the purpose of coercion, punishment, intimidation, or for a discriminatory reason, by a state official or with state consent or acquiescence [28]. In the cases reviewed, it is apparent that U.S. officials intentionally carried out actions causing severe pain and suffering in order to punish, coerce, and intimidate mainly Central American asylum seekers to not pursue their asylum claims. Torture and cruel, inhumane, and degrading treatment are violations of human rights and are prohibited under domestic and international law in any and all circumstances [28].

Given the severity of psychological distress and impairment caused by forced separation, which persist even following reunification, parents and children require professional mental health support in a supportive community environment in order to rehabilitate and recover functionality. The clinicians noted that recovery most likely will not occur in the following scenarios: 1) detention settings, 2) ongoing forced separation from family members, 3) forced return to their country of origin, and 4) re-exposure to pre-migration trauma. Community-based settings in which families can safely access social support such as friends, family and religious communities, as well as work and school, are the most appropriate environment for recovery. Furthermore, functional impairment may continue to impede individuals’ ability to participate in their immigration case, absent significant accommodations such as breaks, utilizing simple language, and rephrasing questions as needed.

## Limitations

This study has several limitations. Firstly, the data was not collected for research purposes and therefore was not uniform in structure. The population captured in our analysis was not selected in a systematic manner and may be unique in that they all had legal representation. Nevertheless, the rich and illustrative narratives of this cohort help shed light on the experiences of separated families [29, 30].

No children under the age of six were represented in the dataset, so the impacts of separation on infants and toddlers is not assessed in this study. Most of the families included in this data set were separated for an average of 30–70 days. Families who were separated for much longer may have experienced even greater negative health consequences as a result of the separation. Thus, further study is needed regarding the impact of separation in populations with especially prolonged separation. Finally, the materials analyzed are narrative reports of clinicians who interviewed survivors and are not direct transcriptions of interviews with the affected individuals. As such, reporting bias is a limitation.

## Conclusion

Untreated trauma can have chronic and long-lasting effects on both adults and on children and adversely affect their physical, mental, developmental, and behavioral health. Those who experience trauma, especially as children, have higher rates of chronic medical conditions such as cardiovascular disease, cancer, and premature death [8]. In addition, there is an increased risk of psychiatric disorders such as anxiety, depression, and psychosis, and of detrimental coping behaviors such as smoking and use of alcohol or drugs [18, 19, 31, 32]. Recovery from trauma is possible, but requires: 1) avoidance of re-traumatization, 2) psychiatric and behavioral health interventions, 3) strong social and family-mediated support.

The decision to separate very young children, including nursing and preverbal children, from their parents, without any intent to reunify or even to effectively track the separations, is not a legitimate policy choice—indeed the policy violated well-established principles of human rights. The resulting severe psychological harms, which were intentionally inflicted by the U.S. government, creates a moral imperative to assist this population, and to do so urgently.

Parents who have been deported, their children still suffering deeply in the United States, must be reunified in the United States. Culturally responsive trauma-informed mental health support must be provided [33, 34]. Accommodations—such as offering breaks, using simple language and translations, providing additional time to process what is being asked—should be available to ensure a trauma-informed adjudication process. One Guatemalan mother, at the end of her evaluation, asked the psychologist if there was any way to forget about the trauma of separation because it is so painful for her. This question must be answered with multi-system, multi-sectoral support, including health and mental health services, legal services, and thoughtful reparations for the harm caused by this policy. Families should be consulted regarding the most appropriate means of reparation [35–37], including but not limited to a formal apology by the U.S. government, a pathway to permanent legal residence, monetary damages and prosecution of the officials who implemented this policy. The policy of forced family separation should also be a catalyst to actions to create a more humane immigration system which can prevent such trauma in the future. Clinicians and health professionals can articulate the pressing need for a trauma-informed immigration system in a powerful way through their expertise [38]. The authoritative voice of clinicians in documenting trauma and advocating for policy change is part of a global movement which recognizes immigration policies as a critical structural determinant of health [39–41].

## Supporting information

S1 File IRB family separation. (PDF)

## References

[1] Dickerson C. The youngest child separated from his family at the border was 4 months old. The New York Times. June. 2019 Jun 16;16.

[2] Trump administration was warned of “traumatic psychological injury” from family separations, official says [Internet]. PBS NewsHour. 2018 [cited 2021 Oct 9]. Available from: https://www.pbs.org/newshour/politics/trump-administration-was-warned-of-traumatic-psychological-injury-from-family-separations-official-says

[3] Letter to Chairman Grassley and Vice Chairman Wyden, Senate Whistleblowing Caucus, July 17, 2018, https://www.whistleblower.org/wp-content/uploads/2019/01/Original-Docs-Letter.pdf.

[4] Justice Department IG report on family separations—CNNPolitics [Internet]. [cited 2021 Oct 9]. Available from: https://www.cnn.com/2021/01/14/politics/doj-report-family-separations/index.html

[5] Interagency Task Force on the Reunification of Families. Interim Progress Report. Homeland Security. 2021 Aug. [cited 2021 Oct 9]. Available from: https://www.dhs.gov/sites/default/files/publications/21_0826_s1_interim-progress-report-family-reunification-task-force.pdf.

[6] Woodward C, Yen H, and Alonso-Zaldivar R. AP FACT CHECK: Falsehoods and fumbles in Trump-Biden debate [Internet]. AP NEWS. 2021 [cited 2021 Oct 9]. Available from: https://apnews.com/article/ap-fact-check-final-debate-trump-biden-4d304cf7ce7dee9c228f48bd9b76e8f7

[7] Oral R, Ramirez M, Coohey C, Nakada S, Walz A, Kuntz A, et al. Adverse childhood experiences and trauma informed care: the future of health care. Pediatric research. 2016 Jan;79(1):227–33. doi: 10.1038/pr.2015.197 26460523

[8] Felitti VJ, Anda RF, Nordenberg D, Williamson DF, Spitz AM, Edwards V, et al. Relationship of childhood abuse and household dysfunction to many of the leading causes of death in adults: The adverse childhood experiences (ACE) study. American journal of preventive medicine. 2019 Jun. doi: 10.1016/j.amepre.2019.04.001 9635069

[9] Oh DL, Jerman P, Marques SS, Koita K, Ipsen A, Purewal S, et al. Systematic review of pediatric health outcomes associated with adverse childhood experiences (aces). doi: 10.1016/j.amepre.2018.11.030 30905481

[10] Lê-Scherban F, Wang X, Boyle-Steed KH, Pachter LM. Intergenerational associations of parent adverse childhood experiences and child health outcomes. Pediatrics. 2018 Jun 1;141(6). doi: 10.1542/peds.2017-4274 29784755

[11] Keller A, Joscelyne A, Granski M, Rosenfeld B. Pre-migration trauma exposure and mental health functioning among Central American migrants arriving at the US border. PLoS One. 2017 Jan 10;12(1):e0168692. doi: 10.1371/journal.pone.0168692 28072836 PMC5224987

[12] Keller AS, Rosenfeld B, Trinh-Shevrin C, Meserve C, Sachs E, Leviss JA, et al. Mental health of detained asylum seekers. The Lancet. 2003 Nov 22;362(9397):1721–3. doi: 10.1016/S0140-6736(03)14846-5 14643122

[13] Steel Z, Silove D, Brooks R, Momartin S, Alzuhairi B, Susljik IN. Impact of immigration detention and temporary protection on the mental health of refugees. The british journal of psychiatry. 2006 Jan;188(1):58–64. doi: 10.1192/bjp.bp.104.007864 16388071

[14] Teicher MH. Childhood trauma and the enduring consequences of forcibly separating children from parents at the United States border. BMC medicine. 2018 Dec;16(1):1–3. 10.1186/s12916-018-1147-y PMC6103973 30131056

[15] Wise J. American Academy of Pediatrics president: Trump family separation policy is “child abuse” | TheHill [Internet]. [cited 2021 Oct 9]. Available from: https://thehill.com/latino/392790-american-academy-of-pediatrics-president-trumps-family-separation-policy-is-child.

[16] Habbach H, Hampton K, Mishori R. You Will Never See Your Child Again [Internet]. Physicians for Human Rights. [cited 2021 Oct 9]. Available from: https://phr.org/our-work/resources/you-will-never-see-your-child-again-the-persistent-psychological-effects-of-family-separation/

[17] MacLean SA, Agyeman PO, Walther J, Singer EK, Baranowski KA, Katz CL. Mental health of children held at a United States immigration detention center. Social Science & Medicine. 2019 Jun 1;230:303–8. 31047760 10.1016/j.socscimed.2019.04.013

[18] Geltman PL, Grant-Knight W, Mehta SD, Lloyd-Travaglini C, Lustig S, Landgraf JM, et al. The “lost boys of Sudan”: Functional and behavioral health of unaccompanied refugee minors resettled in the United States. Archives of pediatrics & adolescent medicine. 2005 Jun 1;159(6):585–91. 15939860 10.1001/archpedi.159.6.585

[19] Hodes M. Psychopathology in refugee and asylum-seeking children. In: Rutter’s child and adolescent psychiatry, 5th ed. Wiley Blackwell; 2008. p. 474–86.

[20] Bronstein I, Montgomery P. Psychological distress in refugee children: a systematic review. Clinical child and family psychology review. 2011 Mar;14(1):44–56. doi: 10.1007/s10567-010-0081-0 21181268

[21] Miller A, Hess JM, Bybee D, Goodkind JR. Understanding the mental health consequences of family separation for refugees: Implications for policy and practice. American journal of orthopsychiatry. 2018;88(1):26. doi: 10.1037/ort0000272 28617002 PMC5732089

[22] Bean T, Derluyn I, Eurelings-Bontekoe E, Broekaert E, Spinhoven P. Comparing psychological distress, traumatic stress reactions, and experiences of unaccompanied refugee minors with experiences of adolescents accompanied by parents. The Journal of nervous and mental disease. 2007 Apr 1;195(4):288–97. doi: 10.1097/01.nmd.0000243751.49499.93 17435478

[23] Corbin J, Strauss A. Basics of qualitative research: Techniques and procedures for developing grounded theory. Sage publications; 2014 Nov 25.

[24] Kelle U. " Emergence" vs." forcing" of empirical data? A crucial problem of" grounded theory" reconsidered. Historical Social Research/Historische Sozialforschung. Supplement. 2007 Jan 1:133–56.

[25] Hennink MM, Kaiser BN, Marconi VC. Code saturation versus meaning saturation: how many interviews are enough? Qualitative health research. 2017 Mar;27(4):591–608. doi: 10.1177/1049732316665344 27670770 PMC9359070

[26] Van Schaack B. Father-Son Separation at US Border Illustrates Lasting Harm That Demands Redress [Internet]. Just Security. 2021 [cited 2021 Oct 9]. Available from: https://www.justsecurity.org/74079/father-son-separation-at-us-border-illustrates-lasting-harm-that-demands-redress/

[27] USA immigration policies resulted in more family separations than disclosed [Internet]. Amnesty International. 2018 [cited 2021 Oct 9]. Available from: https://www.amnesty.org/en/latest/press-release/2018/10/usa-treatment-of-asylum-seekers-southern-border/

[28] Assembly UG. Article 1. Convention against torture and other cruel, inhuman or degrading treatment or punishment. United Nations, Treaty Series. 1984 Dec 10;1465:85.

[29] Payne G, Williams M. Generalization in qualitative research. Sociology. 2005 Apr;39(2):295–314.

[30] Crouch M, McKenzie H. The logic of small samples in interview-based qualitative research. Social science information. 2006 Dec;45(4):483–99.

[31] Bronstein I, Montgomery P. Psychological distress in refugee children: a systematic review. Clinical child and family psychology review. 2011 Mar;14(1):44–56. 10.1007/s10567-010-0081-0 21181268

[32] Miller A, Hess JM, Bybee D, Goodkind JR. Understanding the mental health consequences of family separation for refugees: Implications for policy and practice. American journal of orthopsychiatry. 2018;88(1):26. doi: 10.1037/ort0000272 28617002 PMC5732089

[33] Gartland MG, Hidalgo JA, Danaher FS. Case 20–2020: A 7-Year-Old Girl with Severe Psychological Distress after Family Separation. N Engl J Med. 2020 Jun 25;382(26):2557–65. doi: 10.1056/NEJMcpc2002413 32579817

[34] Paskey S. Telling Refugee Stories: Trauma, Credibility, and the Adversarial Adjudication of Claims for Asylum. Santa Clara Law Review. 2016 Jan 1;56:457–530.

[35] Ford M. The Case for Migrant Reparations. The New Republic [Internet]. 2018 Aug 1 [cited 2021 Oct 9]; Available from: https://newrepublic.com/article/150348/case-migrant-reparations

[36] Opinion | The U.S. owes separated migrant families much more than reunification—The Washington Post [Internet]. [cited 2021 Oct 9]. Available from: https://www.washingtonpost.com/opinions/2021/08/31/separated-migrant-families-reunification-resources/

[37] Families separated at Mexico border ask for residency, aid [Internet]. AP NEWS. 2021 [cited 2021 Oct 9]. Available from: https://apnews.com/article/lifestyle-mexico-immigration-838f4b54515aeff87cc2ec67097c56ee

[38] Physicians for Human Rights Asylum Policy Working Group. Re-Imagining the Asylum System: Recommendations from Asylum Medicine Experts | Health Affairs Blog [Internet]. [cited 2021 Oct 9]. Available from: https://www.healthaffairs.org/do/10.1377/hblog20210510.133971/full/

[39] Dudley M, Young P, Newman L, Gale F, Stoddart R. Health professionals confront the intentional harms of indefinite immigration detention: an Australian overview, evaluation of alternative responses and proposed strategy. International Journal of Migration, Health and Social Care. 2020 Jan 1;17(1):35–51.

[40] Essex RW. Australian Immigration Detention: How Should Clinicians Respond? [Internet] [Thesis]. 2019 [cited 2021 Oct 9]. Available from: https://ses.library.usyd.edu.au/handle/2123/20642

[41] Castañeda H, Holmes SM, Madrigal DS, Young M-ED, Beyeler N, Quesada J. Immigration as a social determinant of health. Annu Rev Public Health. 2015 Mar 18;36:375–92. doi: 10.1146/annurev-publhealth-032013-182419 25494053

