# Supplementary material for: The psychological effects of forced family separation on asylum-seeking children and parents at the US-Mexico border: A qualitative analysis of medico-legal documents
Source: PLoS One. 2021 Nov 24;16(11):e0259576. doi: 10.1371/journal.pone.0259576 (PMC8612557; doi:10.1371/journal.pone.0259576)
Supplement: S1 File — (PDF) [file pone.0259576.s001.pdf]

UNIVERSITY OF MICHIGAN
eResearch.umich.edu

To: Mary Ellen Heisler

Cc:

Mary Ellen Heisler

Subject: eResearch System-Generated Notice of "Not Regulated" Status for HUM00160477

SUBMISSION INFORMATION
Title: Review of De-Identified Medical Affidavits of US Asylum Seekers who Experienced Family Separation
Full Study Title (if applicable): Review of De-Identified Medical Affidavits of US Asylum Seekers who Experienced Family Separation
Study eResearch ID: HUM00160477
Date of this System-Generated Notice: 3/5/2019

IRB "NOT REGULATED" STATUS:

Based on the information provided, the proposed study does not fit the definition of research involving human subjects (45CFR46.102) because the researchers intending to contribute to generalizable knowledge do not interact with human subjects, nor obtain identifiable private information or identifiable biospecimens.
